# Scarcity and Cooperation: The Modulation of Social Norms

**DOI:** 10.3390/bs15070913

**Published:** 2025-07-04

**Authors:** Qiuling Luo, Changjin Qiu, Sihan Dong, Ronghui Tang, Chunhua Kang

**Affiliations:** 1 Zhejiang Philosophy and Social Science Laboratory for the Mental Health and Crisis Intervention of Children and Adolescents, Zhejiang Normal University, Jinhua 321000, China; qiuchangjin@zjnu.edu.cn (C.Q.); dongsihann@zjnu.edu.cn (S.D.); 2 Zhaoqing City Hengli Senior High School, Zhaoqing 526000, China; tangronghui2025@163.com

**Keywords:** scarcity mindset, social norms, descriptive norms, injunctive norms, cooperation

## Abstract

Given the continued relevance of perceived scarcity, understanding how a scarcity mindset influences human cooperation remains critical. However, previous research has yielded mixed results regarding this relationship. To clarify these inconsistencies, this study examined the impact of a scarcity mindset on cooperation within various social normative contexts. Participants were randomly assigned to either a scarcity or abundance mindset and engaged in a Public Goods Game under descriptive or injunctive normative conditions, each with high or low prosocial expectations. The results revealed that in both normative contexts, individuals with a scarcity mindset exhibited greater cooperation than those with an abundance mindset. Individuals also cooperated more under high prosocial norms compared to low ones. Importantly, the interaction effect revealed that while cooperation did not differ between the two mindsets under low prosocial norms, the scarcity mindset led to significantly greater cooperation under high prosocial norms. These findings provide new insights into the scarcity–cooperation dynamic and suggest that robust prosocial norms can amplify the cooperative tendencies associated with a scarcity mindset, highlighting the importance of leveraging social norms to enhance cooperation in resource-scarce situations.

## 1. Introduction

Resource scarcity remains a challenge in many regions, particularly in access to essential natural resources such as water, minerals, and food (He et al., 2021; Maxwell & Reuveny, 2000). In some cases, such scarcity has contributed to regional and international tensions (Colgan, 2011; Kilgour et al., 2020). Similarly, limited access to social resources, including quality education, liquid capital, and employment opportunities, can also heighten individuals’ perceived threat and urgency (DeVoe & Pfeffer, 2011; Zhu et al., 2019). These challenges may not solely stem from objective shortages but also from the psychological impact of perceived scarcity, often referred to as the ‘scarcity mindset’ (Agneman et al., 2023). Mani and colleagues argue that scarcity is a relative concept, referring to a state in which an individual’s resources are insufficient to meet their survival and development needs (Mani et al., 2013; Sharma & Alter, 2012). This scarcity mindset is increasingly recognized as a relevant factor in modern life, subtly shaping individuals’ thoughts and actions (Chou et al., 2016; Krosch & Amodio, 2019; Liang et al., 2021; Petersen et al., 2014; Zhao & Tomm, 2018).

Recent psychological research has highlighted the complex relationship between perceived resource scarcity and prosocial behavior (Agneman et al., 2023; Nie et al., 2020; Petersen et al., 2014). However, the question of whether scarcity mindsets enhance prosocial tendencies remains unresolved. On one hand, some evidence suggests that the urgency of physiological needs may impair certain forms of prosocial behavior. For instance, individuals in a scarcity mindset have been observed to exhibit lower levels of cooperation, a behavior characterized by mutual benefit, compared to those with an abundance mindset (Roux et al., 2015; Tan et al., 2024). Similarly, Agneman et al. (2023) observed that farmers displayed reduced interpersonal trust during poor harvest seasons compared to more abundant periods, which may reflect a broader decline in cooperative tendencies under conditions of scarcity. On the other hand, research has also found that scarcity may encourage prosocial behavior under certain conditions (Faber & Häusser, 2022). Research on socioeconomic status indicates that individuals from lower social classes are often more generous, charitable, and trusting than those in higher social classes (Huijsmans et al., 2019; Piff et al., 2010). Studies on water scarcity show that such condition can foster cooperation in agricultural settings, where interdependence is essential (Gneezy et al., 2016; Nie et al., 2020).

Taken together, the literature presents a nuanced and sometimes contradictory picture of the relationship between scarcity and prosociality (Faber & Häusser, 2022; Piff et al., 2010; Roux et al., 2015). Research has identified several individual differences in personality traits and emotional states that may modulate this relationship (Bernhard et al., 2006; Bonan et al., 2020; Faber & Häusser, 2022; Salerno & Escoe, 2020; Tang et al., 2022; Trollman et al., 2021). For instance, cognitive empathy has been shown to mitigate the negative effect of scarcity on prosociality at both behavioral and neural levels (Alonso-Ferres et al., 2020; Cui et al., 2022; Liu et al., 2023; Li et al., 2023). However, few studies have explored macro-level moderators such as contextual factors that might explain when and why scarcity mindset either promotes or inhibits prosocial behavior (Bartoš, 2021; Civai et al., 2022, 2024; Gelfand et al., 2011). Addressing this gap would deepen our theoretical understanding of how scarcity mindset shapes social decision-making.

One such contextual factor is the presence of social norms, which guide behavior by signaling what others typically do or approve of (Bernhard et al., 2006; Bonan et al., 2020). Research consistently shows that people are more likely to cooperate under prosocial normative conditions, reflecting a strong tendency to conform to collective behavioral standards (Fehr & Schurtenberger, 2018; Hackel et al., 2020). Research has implied a broad link between scarcity mindset and norm adherence. For instance, a theoretical model by Nhim et al. (2019) posits that scarcity can hinder cooperation, especially when inequality leads poorer landowners to mimic the selfish behaviors of wealthier peers. Similarly, a lab-in-the-field study found that farmers in tight-knit communities were less likely to punish unfairness during lean season (scarcity) compared to post-harvest season (abundance), possibly because maintaining cooperative norms is more critical in times of limited resources (Bartoš, 2021). However, these studies have not formally defined or manipulated social norms, nor have they systematically investigated how scarcity interacts with normative cues to shape prosocial behavior. Gaining clarity on this interaction could provide valuable insight into human adaptations to resource scarcity. We might predict that individuals with a scarcity mindset may be more susceptible to normative influence on prosocial behavior than those with an abundance mindset.

An unresolved issue in this domain concerns the differential effects of normative types. Drawing on the Integrative Model of Behavioral Prediction (IM; Fishbein & Yzer, 2003), social norms are fundamentally categorized as descriptive or injunctive (Reno et al., 1993; Stok & de Ridder, 2019; van Kleef et al., 2019). Descriptive norms, or conventions, refer to behaviors that are “typically done”, offering individuals with information about the actions or beliefs of others (Cialdini et al., 1990). Such information often leads individuals to adjust their behaviors accordingly (Bicchieri & Xiao, 2009; Cialdini & Goldstein, 2004). In contrast, injunctive norms, or prescriptive norms, represent behaviors that are ‘socially approved’ and specify what is expected in certain situations (van Kleef et al., 2019). These norms are reinforced by social rewards for adherence and sanctions for deviance. By signaling what is consider ‘correct’, injunctive norms encourage individuals to adjust their behavior to meet societal expectations (McAuliffe et al., 2017; Versluis & Papies, 2016). While prior research has demonstrated that both norm types can promote prosocial behavior, the extent to which they modulate such behavior under conditions of scarcity remains unclear.

To address these questions, three experiments were conducted to investigate how mindset influences prosocial behavior under varying normative contexts. Experiments 1 (N = 60) and 2 (N = 54) examined the effects of descriptive norms, while Experiment 3 focused on injunctive norms (N = 70). Unlike previous studies that manipulated norms through explicit verbal instructions (Jacobson et al., 2021; Kredentser et al., 2012), the present study allowed participants to infer descriptive and injunctive norms indirectly through interactive contextual cues, such as the proportion of cooperative group members and the structure of group-based incentives. Based on prior findings, we developed the following hypotheses: First, participants with a scarcity mindset, compared to those with an abundance mindset, will demonstrate greater prosocial behavior when prosocial norms are present (H1). Second, the moderating effect of social norms on the relationship between mindset and prosocial behavior will be significant for both descriptive and injunctive norms (H2). All research materials and data are publicly available at the following link: https://osf.io/v3a9x/?view_only=a0031efe55144e1bbc6652b89c917f12 (accessed 16 November 2024).

## 2. Experiment 1: The Impact of Descriptive Norms on Cooperation Rate

### 2.1. Methods

#### 2.1.1. Participants

The experiment initially employed G*Power (version 3.1; Faul et al., 2009) for sample size estimation. With a significance level of α = 0.05 and an effect size of *f* = 0.25, the required sample size required to achieve 95% statistical power was estimated to be 54. Consequently, 60 undergraduate students (46 females; M_age_ = 18.42 years, SD = 0.97; Han nationality) were recruited. Participants were randomly assigned to either the scarcity mindset group or the abundance mindset group.

- Scarcity mindset group: 30 students (M_age_ = 18.20 years, SD = 0.48; 22 females)
- Abundance mindset group: 30 students (M_age_ = 18.63 years, SD = 1.27; 24 females)

All participants were required to be healthy, right-handed, with normal or corrected-to-normal vision, no history of mental or neurological disorders, and had not previously participated in similar experimental studies. A sensitivity power analysis using G*Power indicated that the sample size of 60 participants provided 80% power to detect an effect size of *f* = 0.184 or greater in a repeated measures ANOVA with α = 0.05. This experiment received approval from the university’s Human Research Ethics Committee. All participants provided informed consent prior to the experiment and were compensated for their participation.

#### 2.1.2. Design and Procedure

The present study utilized a 2 (mindset: scarcity vs. abundance)  ×  2 (descriptive norms: high prosocial vs. low prosocial) mixed-factorial design, with mindset as the between-subject factor and descriptive norms as the within-subjects factor. Cooperative rate in the Public Goods Game served as the dependent variable.

**Figure 1:**
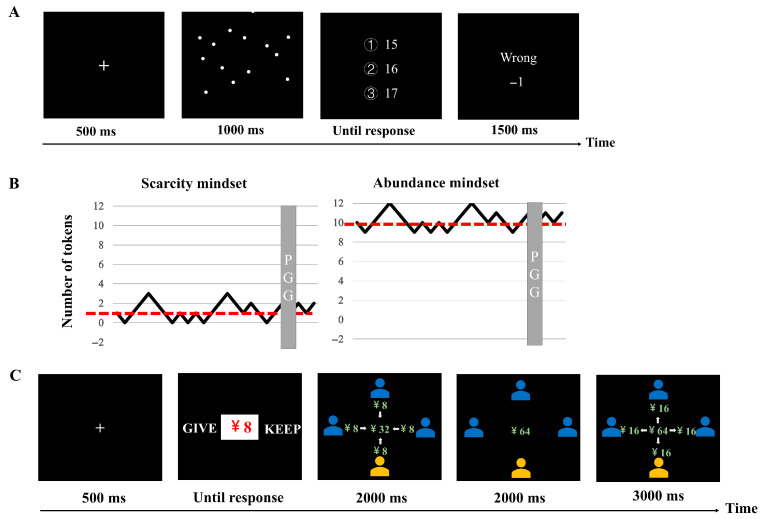
The experiment design. (**A**) Illustrations of the perceptual judgment task. (**B**) The experimental structure. The red dashed lines indicate the threshold, while the black lines represent the variation in the number of tokens around the threshold under scarcity (**left** panel) and abundance mindsets (**right** panel). The gray shaded areas highlight the main task stages. (**C**) A flowchart illustrating the main task (i.e., the PGG).

Upon arrival, each participant was randomly assigned to one of the two mindset conditions. The scarcity and abundance mindsets were induced through a perceptual judgment task (PJT; Huijsmans et al., 2019), comprising two blocks of 30 trials each. Participants were required to estimate the number of dots displayed on the screen (Figure 1A). To induce a scarcity mindset, participants started with one token, and their token count remained at approximately one throughout the experiment. In contrast, the abundance mindset was induced by giving participants 10 initial tokens, with their token count fluctuating around 10 (Figure 1B). Feedback was provided after each keypress, and the current token count was shown at the end of the PJT. All participants were informed that they needed to retain at least one token at the end of each block to earn a 5 RMB reward.

The Public Goods Game (PGG) was intertwined with the PJT. During the interval of PTJ, participants engaged in the PGG. In each trial of the PGG, participants were grouped with three other players, each receiving an initial 8 RMB. They decided whether or not to contribute all their money to a public pool, which would be doubled and equally distributed among all four players. After each trial, contributions and earnings of all players were displayed. The average earnings across all trials determined each participants’ final earnings. Participants were informed that they would not be paired with the same players twice, ensuring one-short interactions (Figure 1C).

The PGG consisted of two blocks—high prosocial and low prosocial—each with 25 trials. In the high prosocial block, participants were paired with 75 players (three players per trial), 52 (70%) of whom contributed all of their tokens. In this block, 1 trial featured no contributions from any of the three players, 5 trials had one player contributing all tokens, 10 trials had two players contributing all tokens, and 9 trials had all three players contributing. In the low prosocial block, 23 (30%) of the 75 players contributed all their tokens. This block included 9 trials where none of the three players contributed all tokens, 10 trials with one player contributing all tokens, 5 trials with two players contributing, and 1 trial with all players contributing. Following each block of the PGG, participants’ perception of social norm was measured by scoring the averaged cooperation rate of paired players on a scale from 0 to 100%.

Furthermore, to assess the effectiveness of scarcity and abundance mindset manipulation (Huijsmans et al., 2019), participants were asked to evaluate subjective ratings of their confidence, stress, motivation, and excitement using a seven-point Likert scale, with higher scores indicating greater levels of each rating. The assessments were collected after the completion of all tasks.

### 2.2. Results

Data analysis and processing were conducted using SPSS 27.0 software.

#### 2.2.1. Manipulation Checks for Mindset and Descriptive Norms

An independent-samples *t*-test was conducted to confirm the effectiveness of the mindset manipulation. Results showed that participants in the scarcity mindset reported significantly lower confidence (*t*(58) = 3.55, *p* < 0.001, Cohen’s *d* = 0.86) and higher stress (*t*(58) = −3.98, *p* < 0.001, Cohen’s *d* = 1.01) compared to those in the abundance mindset. No significant differences were found between these two mindsets regarding motivation or excitement; *ps* ≥ 0.39.

Next, to test the effectiveness of the descriptive norms manipulation, a paired-samples *t*-test was conducted on participants’ norm perception (i.e., cooperation rate of paired players). Results showed that participants perceived significantly higher cooperation in the high prosocial condition (72.38% ± 13.9%) compared to the low prosocial condition (29.7% ± 18.0%), *t*(59) = 17.05, *p* < 0.001, Cohen’s *d* = 2.20, confirming the effectiveness of the descriptive norms manipulation.

#### 2.2.2. Effects of Mindset and Descriptive Norms on Cooperation Rate

A 2 (mindset: scarcity vs. abundance) × 2 (descriptive norms: high prosocial vs. low prosocial) mixed ANOVA was conducted on cooperation rate (the percentage of “GIVE” options). The results showed a significant main effect of mindset—*F*(1, 58) = 3.99, *p* = 0.05, η_p_^2^ = 0.06—with higher cooperation in the scarcity mindset (40.53% ± 21.03%) than in the abundance mindset (30.27% ± 18.72%). A significant main effect of descriptive norms also emerged—*F*(1, 58) = 88.63, *p* < 0.001, η_p_^2^ = 0.60—with higher cooperation in the high prosocial condition (48.40% ± 2.99%) than the low prosocial condition (22.40% ± 19.36%).

**Figure 2:**
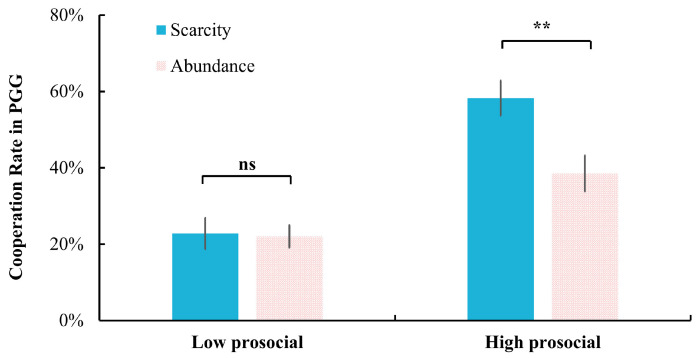
The effect of mindset on cooperation rate in PGG under high and low prosocial descriptive norms. **: *p* < 0.01; ns: not significant, *p* > 0.05.

The interaction between mindset and descriptive norms was significant: *F*(1, 58) = 11.75, *p* < 0.001, η_p_^2^ = 0.17. Simple effects analysis revealed that under the high prosocial condition, the scarcity mindset (58.27% ± 24.98%) led to significantly higher cooperation compared to the abundance mindset (38.53% ± 25.61%), *p* = 0.004. However, under the low prosocial condition, no significant difference was detected between the scarcity mindset (22.80% ± 22.41%) and the abundance mindset (22.00% ± 16.13%), *p* = 0.874 (Figure 2).

#### 2.2.3. Effects of Mindset and Descriptive Norms on Deviation from Social Norm

The averaged cooperation rate of other players was used to establish the cooperation norm standard—70% in the high prosocial condition and 30% in the low prosocial condition. Norm deviation was calculated by subtracting the group’s averaged cooperation rate from each participant’s cooperation rate. This measure indicates the extent to which participants’ behavior aligns with the social cooperation norm.

A 2 (mindset) × 2 (descriptive norms) mixed ANOVA was conducted on norm deviation. A significant main effect of mindset was found, *F*(1, 58) = 3.99, *p* = 0.050, η_p_^2^ = 0.06. Participants in the scarcity mindset (−9.47% ± 21.03%) deviated less from the norm compared to those in the abundance mindset (−19.73% ± 18.72%). A significant main effect of descriptive norms was also reported, *F*(1, 58) = 25.70, *p* < 0.001, η_p_^2^ = 0.31, with less deviation under the low prosocial condition (−7.60% ± 19.36%) than high prosocial condition (−21.60% ± 26.99%).

**Figure 3:**
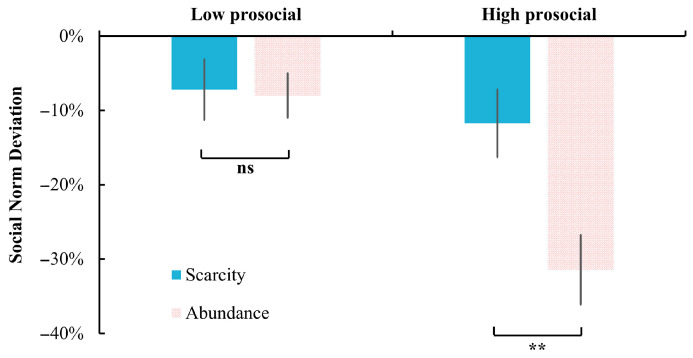
The effect of mindset on social norm deviation under high and low prosocial descriptive norms in Experiment 1. **: *p* < 0.01; ns: not significant, *p* > 0.05.

The interaction effect between mindset and descriptive norms was also significant, *F*(1, 58) = 11.75, *p* = 0.001, η_p_^2^ = 0.17. Further analysis revealed that under the high prosocial condition, participants in the scarcity mindset (−11.73% ± 24.98%) deviated less compared to those in the abundance mindset (−31.47% ± 25.61%), *p* = 0.004. No difference was found under the low prosocial condition (scarcity: −7.20% ± 22.41%; abundance: −8.00% ± 16.13%), *p* = 0.874 (Figure 3).

#### 2.2.4. Effects of Mindset and Social Norms on Norm Perception

To figure out whether mindset influences individuals’ perception of norms, independent-samples *t*-tests were conducted respectively for high and low prosocial conditions. No significant differences in norm perception were found between mindsets in either the high prosocial (scarcity: 71.07% ± 12.33%; abundance: 71.70% ± 15.51; *p* = 0.862) or low prosocial conditions (scarcity: 29.63% ± 19.16%; abundance: 29.77% ± 17.04%; *p* = 0.977). Thus, mindset did not affect participants’ ability to perceive descriptive norms.

### 2.3. Discussion

Experiment 1 examined the impact of mindset on individual cooperation within a descriptive normative. Participants exposed to high prosocial norms exhibited significantly greater cooperation than those in the low prosocial condition, consistent with previous findings. Descriptive norms, conceptualized as perceptions of the prevalence of a behavior within a referent group, are shaped through behavioral observation and interpersonal communication (Cialdini et al., 1990; Fehr & Gächter, 2002). Previous research have demonstrated that such norms can produce strong conformity, sometimes even taking precedent over individual preferences (Irwin & Simpson, 2013; Rimal, 2008; Schultz et al., 2007). In the current study, participants under high prosocial norms were exposed to a reference group in which most members contributed generously, likely guiding their own decisions to cooperate. These findings suggests that strong descriptive norms, where the majority of participants behave cooperatively, can exert a substantial influence on individuals’ cooperation.

Importantly, individuals with a scarcity mindset cooperated at higher rates than those with an abundance mindset, aligning with previous research (Nie et al., 2020; Osman et al., 2018). This supports the notion that scarcity can heighten sensitivity to group welfare, especially when prosocial behavior is socially endorsed (Nie et al., 2020; Osman et al., 2018). For example, during the COVID-19 pandemic, increased perceived scarcity of social connection predicted a greater cooperation in public goods game (Civai et al., 2022). Similarly, a large-scale cross-culture study reported that chronic subjective experiences of scarcity were associated with higher scores in several morality measures, suggesting increased prosocial tendencies (Elbæk et al., 2023). Our findings suggest that a scarcity mindset may activate a heightened sensitivity to social cues, making individuals more likely to align their behavior with prevailing social norms.

A critical interaction further supports this interpretation: under the high prosocial condition, participants with a scarcity mindset exhibited significantly greater cooperation compared to those with an abundance mindset. However, no such differences emerged under the low prosocial condition. This finding was consistent with Hypothesis 1, suggesting that scarcity enhances sensitivity to strongly prosocial expectations but not to weak or ambiguous norms. When normative cues are unclear, mindset appears to exert less influence on cooperative behavior. Supporting this, norm deviation analysis revealed that those in a scarcity mindset adhered more closely to the normative standard in the high prosocial condition. Thus, the effects of scarcity on cooperation appear highly context-dependent, emerging predominantly when normative expectations are clear and compelling.

An alternative explanation is that mindset may alter individuals’ norm perception, which in turn regulates their subsequent cooperative behavior. However, analysis showed no significant differences in norm perception between scarcity and abundance mindsets, ruling out this possibility. The interaction effect is therefore more likely due to differences in norm adherence rather than perception. Lastly, it is important to acknowledge a limitation: cooperation in this experiment was measured as a binary choice, limiting our ability to capture nuanced degrees of cooperative intent. To address this, Experiment 2 will adopt a continuous contribution paradigm, allowing for more nuanced evaluation and validation of the current findings.

## 3. Experiment 2: The Impact of Descriptive Norms on Cooperative Contribution

### 3.1. Method

#### 3.1.1. Participants

A total of 54 undergraduate students (42 females, M_age_ = 19.22 years, SD = 1.08; Han nationality) were recruited. All participants were healthy and had not participated in experiment 1. Participants were randomly assigned to either the scarcity mindset group or the abundance mindset group.

- Scarcity mindset group: 27 students (M_age_ = 19.04 years, SD = 0.71; 20 females).
- Abundance mindset group: 27 students (M_age_ = 19.41 years, SD = 1.34; 22 females).

A sensitivity power analysis using G*Power indicated that the sample size of 54 participants provided 80% power to detect an effect size of *f* = 0.194 or greater in a repeated measures ANOVA with a 5% false positive rate. This experiment received approval from the university’s Human Research Ethics Committee. All participants provided informed consent prior to the experiment and received monetary compensation for their participation.

#### 3.1.2. Design and Procedure

The experimental design was identical to that of Experiment 1, except that cooperative contribution in the Public Goods Game (PGG) served as the dependent variable. The experimental procedure for Experiment 2 was largely consistent with that of Experiment 1. The key modification was in the Public Goods Game (PGG), where participants were allowed to contribute any amount between ¥0 and ¥8 to a public pool.

### 3.2. Results

Data analysis and processing were conducted using SPSS 27.0 software.

#### 3.2.1. Manipulation Checks for Mindset and Descriptive Norms

An independent-samples *t*-test confirmed the effectiveness of the mindset manipulation. Participants in the scarcity mindset reported significantly lower confidence (*t*(52) = −3.88, *p* < 0.001, Cohen’s *d* = 1.06) and higher stress (*t*(52) = 3.46, *p* = 0.001, Cohen’s *d* = 0.94) than those in the abundance mindset. No significant differences were observed between these two mindsets regarding motivation or excitement; *ps* = 0.084.

A paired-samples *t*-test was conducted on participants’ perceptions of others’ cooperation under high and low prosocial conditions to examine the effectiveness of the norm manipulation. Participants perceived significantly higher cooperation levels under the high prosocial condition (72.19% ± 13.9%) than under the low prosocial condition (36.28% ± 22.4%), *t*(53) = 11.76, *p* < 0.001, Cohen’s *d* = 0.18.

#### 3.2.2. Effects of Mindset and Descriptive Norms on Cooperative Contribution

A 2 (mindset: scarcity vs. abundance) × 2 (descriptive norms: high prosocial vs. low prosocial) mixed ANOVA was conducted on the amount contributed to the public pool in the PGG. A significant main effect of mindset was observed, *F*(1, 52) = 13.65, *p* = 0.001, η_p_^2^ = 0.208, with participants in the scarcity mindset (3.35 ± 1.18) contributed more compared to those in the abundance mindset (2.08 ± 1.35). The main effect of descriptive norms was also significant, *F*(1, 52) = 45.08, *p* < 0.001, η_p_^2^ = 0.464, indicating greater contribution for the high prosocial condition (3.54 ± 2.03) than the low prosocial condition (1.89 ± 1.40).

**Figure 4:**
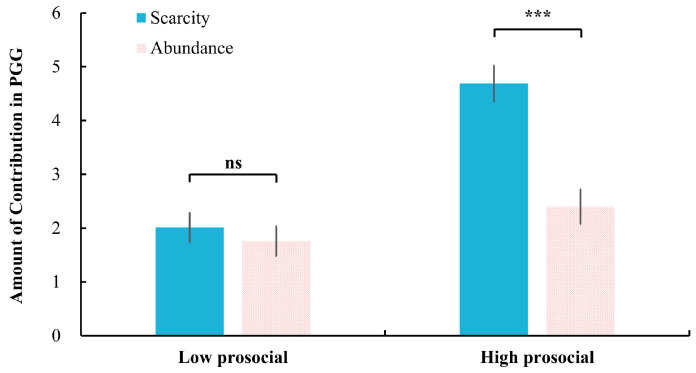
The effect of mindset on the level of contribution in PGG under high and low prosocial descriptive norms. ***: *p* < 0.001; ns: not significant, *p* > 0.05.

Moreover, the interaction effect between mindset and descriptive norms was significant, *F*(1, 52) = 16.95, *p* < 0.001, η_p_^2^ = 0.246. Further analysis showed that in the high prosocial condition, participants in the scarcity mindset (4.69 ± 1.72) contributed significantly more than those in the abundance mindset (2.40 ± 1.66), *p* < 0.001. No significant difference emerged under the low prosocial condition between scarcity (2.01 ± 1.40) and abundance (1.76 ± 1.42) mindsets, *p* = 0.51 (see Figure 4).

#### 3.2.3. Effects of Mindset and Descriptive Norms on Deviation from Social Norms

Using the averaged contributions of other players as the norm benchmark (5.54 for high prosocial and 2.54 for low prosocial conditions), individual norm deviation was calculated by subtracting the group average contribution from the contribution of each participant.

A 2 (mindset) × 2 (descriptive norms) mixed ANOVA Revealed a significant main effect of mindset, *F*(1, 52) = 55.28, *p* < 0.001, η_p_^2^ = 0.21. Participants in the scarcity mindset (−0.69 ± 1.18) deviated less from the norm compared to those in the abundance mindset (−1.96 ± 1.35). There was also a significant main effect of descriptive norms, *F*(1, 52) = 33.58, *p* < 0.001, η_p_^2^ = 0.39, with smaller deviations under the low prosocial condition (−0.65 ± 1.40) than under the high prosocial condition (−2.00 ± 2.03).

**Figure 5:**
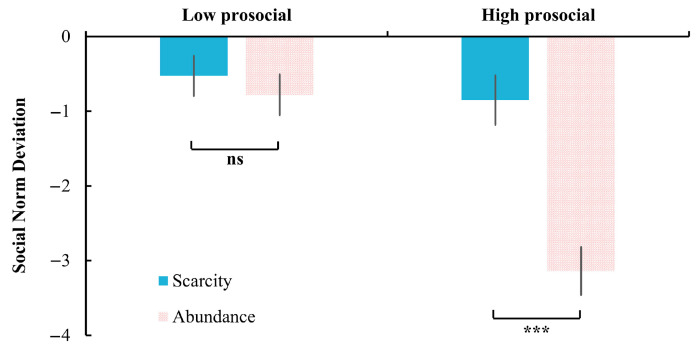
The Effect of Mindset on Social Norm Deviation under High and Low Prosocial Descriptive Norms in Experiment 2. ***: *p* < 0.001; ns: not significant, *p* > 0.05.

The interaction effect was also significant: *F*(1, 52) = 16.95, *p* < 0.001, η_p_^2^ = 0.25; please see Figure 5. Further analysis revealed that in the high prosocial condition, participants in the scarcity mindset (−0.85 ± 1.72) deviated less compared to those in the abundance mindset (−3.14 ± 1.66), *p* < 0.001. However, under the low prosocial condition, there was no significant difference was found between scarcity (−0.53 ± 1.40) and abundance (−0.78 ± 1.42) mindsets, *p* = 0.51.

#### 3.2.4. Effects of Mindset and Social Norms on Norm Perception

Independent-samples *t*-tests were conducted to examine the effect of mindset on norm perception in each prosocial condition. It was found that in the high prosocial condition, no significant difference was found between the scarcity (75.70% ± 13.83%) and abundance (68.67% ± 13.38%) mindsets, *p* = 0.063. Similarly, in the low prosocial condition, norm perception did not differ significantly between the scarcity (32.48% ± 19.15%) and abundance (40.07% ± 25.09%) mindsets, *p* = 0.217. These results suggest that mindset does not influence individuals’ perception of descriptive social norms.

### 3.3. Discussion

Experiment 2 reaffirmed and extended the findings of Experiment 1 by allowing participants to contribute varying amounts in the Public Goods Game, thereby providing a more nuanced measure of cooperation. Consistent with the results from Experiment 1, participants with a scarcity mindset were more cooperative than those with an abundance mindset, aligning with previous findings (Nie et al., 2020; Osman et al., 2018). This pattern reinforces the proposition scarcity heightens individuals’ sensitivity to social cues (Piff et al., 2010).

Moreover, descriptive social norms significantly influenced cooperative behavior. Participants contributed more in the high-prosocial condition than in the low-prosocial condition, highlighting the powerful role of norms in shaping decisions. This aligns with a robust body of literature suggesting that individuals adapt their behavior to match perceived actions of others (Bonan et al., 2020).

Notably, a significant interaction between mindset and norms emerged again, which was also observed in Experiment 1. Participants in the scarcity mindset condition contributed more than those in the abundance mindset, particularly under strong prosocial norms. Further analysis revealed that scarcity-primed individuals deviated less from the normative cooperation level, suggesting that scarcity not only increases overall cooperation but also enhances alignment with social expectations. These findings support the view that a scarcity mindset amplifies adherence to social norms, even when it may not maximize personal gain.

This heightened norm adherence under scarcity resonates with social class research on contextualism. A scarcity mindset simulates the psychological experience of having limited resources, which parallels the situation of lower socioeconomic status. Diminished resources and lower social rank often reduce perceived personal control and promote “contextualist” tendencies—heightened sensitivity to external cues and others’ behaviors (Kraus et al., 2012; Lozano et al., 2020). In the current experiment, scarcity-minded individuals exhibited a strongly contextualist response. They aligned their behavior with the cooperative norm (i.e., high prosocial condition) and resisted the temptation to prioritize self-interest.

Interestingly, the absence of significant differences in the low prosocial norm condition, indicating that the influence of the scarcity mindset on cooperation is contingent upon the strength of social norms. This outcome aligns with prior research showing that uncooperative behavior can quickly spread through groups and suppress others’ willingness to contribute (Fowler & Christakis, 2010). Overall, these results underscore the context-dependent nature of scarcity’s influence on prosocial behavior and highlight the importance of social norms in modulating this relationship.

## 4. Experiment 3: The Impact of Injunctive Norms on Cooperative Contribution

### 4.1. Methods

#### 4.1.1. Participants

A total of 70 undergraduate students were recruited for the experiment (65 females; M_age_ = 19.13 years, SD = 1.35; Han nationality). All participants were healthy, right-handed individuals with normal or corrected-to-normal vision, reported no history of mental or neurological disorders, and no prior participation in similar experimental studies. Participants were randomly assigned to either the scarcity mindset group or the abundance mindset group.

- Scarcity mindset group: 35 students (M_age_ = 18.97 years, SD = 1.15; 33 females).
- Abundance mindset group: 35 students (M_age_ = 19.29 years, SD = 1.53; 32 females).

A sensitivity power analysis using G*Power indicated that the sample size of 70 participants provided 80% power to detect an effect size of *f* = 0.17 or greater in a repeated measures ANOVA with a 5% false positive rate. The study was approved by the university’s Human Research Ethics Committee. All participants provided signed informed consent and were compensated based on their performances.

#### 4.1.2. Design and Procedure

The present study employed a 2 (mindset: scarcity vs. abundance)  ×  2 (injunctive norms: high prosocial vs. low prosocial) mixed-factorial design, with mindset as the between-subject factor. The dependent variable was cooperative contribution in the Public Goods Game (PGG).

Unlike descriptive norms, which are typically learned through observing others’ behaviors, injunctive norms are acquired by inferring which actions are socially approved or disapproved. As documented in previous research, injunctive norms reflect collective evaluations of behavior, often conveyed through the social rewards or sanctions associated with conformity or deviation (Smith & Louis, 2008; White et al., 2009; Yun, 2025). In this sense, reward structures can function as implicit signals of normative approval—indicating not only which behaviors are encouraged, but also which are discouraged within a given group context.

The procedure was similar to that of Experiment 1. Following the approach of Michaeli and Spiro (2018), injunctive norms were manipulated using material incentives. Participants would earn points based on their contributions in the public good game, and these points were later converted to currency at a ratio of 4:1 (4 points = 1 RMB). In the high prosocial condition, a contribution of 1–2 RMB earned 1 point, 3–4 RMB earned 2 points, 5–6 RMB earned 3 points, and 7–8 RMB earned 4 points. In the low prosocial condition, the same contribution ranges earned only 0.1, 0.2, 0.3, or 0.4 points, respectively. After making their decisions, participants were not shown others’ contributions or any trial-by-trial feedback (to avoid introducing descriptive norm information); only their final total earnings were disclosed. Notably, this contribution-based reward system was presented as determined by other group members, serving as a means to manipulate injunctive norms.

Following each block of the PGG, participants were asked to rate the extent to which they felt motivated (or encouraged) by the group for their investment behavior, using a scale from 0 to 100%. In addition, participants reported their expectations regarding other members’ investments and their willingness to join the group. However, as these latter two measures are not directly relevant to the central research question of the current study, they are not included here.

### 4.2. Results

Data analysis and processing were conducted using SPSS 27.0 software.

#### 4.2.1. Manipulation Checks for Mindset and Injunctive Norms

To verify the effectiveness of the experimental manipulations, we conducted manipulation checks for both mindset and injunctive norm conditions. An independent-samples *t*-test revealed that participants in the scarcity mindset reported significantly lower confidence (*t*(52) = −3.88, *p* < 0.001) and higher stress (*t*(52) = 3.46, *p* ≤ 0.001) compared to those in the abundance mindset. These two mindsets did not significantly differ in motivation (*p* > 0.07) or excitement (*p* > 0.09).

Additionally, a paired-samples *t*-test on participants’ perceptions of group motivation (or encouragement) showed that participants perceived significantly higher levels of motivation (or encouragement) under the high prosocial condition (71.63% ± 17.98%) compared to low prosocial condition (37.36% ± 25.01%), *t*(53) = 11.76, *p* < 0.001. This result confirms that the manipulation of injunctive norms was effective.

#### 4.2.2. Effects of Mindset and Injunctive Norms on Cooperative Contribution

A 2 × 2 repeated-measures ANOVA was conducted on participants’ contribution amounts in the PGG, with mindset as the between-subject factor, injunctive norms as the within-subjects factor. A marginally significant effect of mindset was found, *F*(1, 68) = 3.74, *p* = 0.057, η_p_^2^ = 0.05. Participants in the scarcity mindset (4.63 ± 1.34) contributed slightly more compared to those in the abundance mindset (3.97 ± 1.53). The main effect of injunctive norms was significant, *F*(1, 68) = 58.92, *p* < 0.001, η_p_^2^ = 0.46. Contributions in high prosocial condition (5.02 ± 1.68) were significantly higher than in low prosocial condition (3.58 ± 1.71).

**Figure 6:**
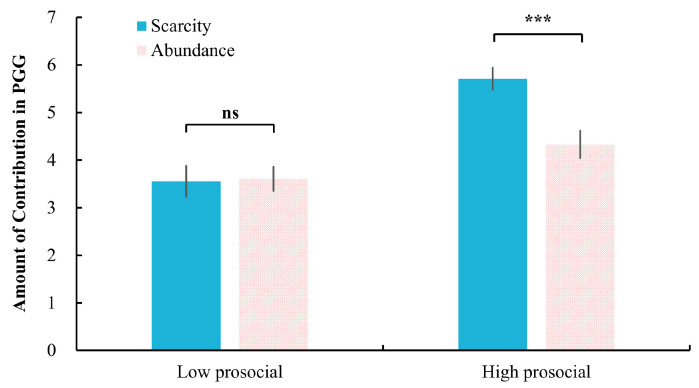
The effect of mindset on the level of contribution in PGG under high and low prosocial injunctive norms. ***: *p* < 0.001; ns: not significant, *p* > 0.05.

The interaction effect between mindset and injunctive norms also reached significance, *F*(1, 68) = 14.65, *p* < 0.001, η_p_^2^ = 0.18. Follow-up comparisons revealed that the under high prosocial condition, participants with a scarcity mindset (5.71 ± 1.38) contributed significantly more than those with an abundance mindset (4.33 ± 1.69), *p* < 0.001. However, under the low prosocial condition, there was no significant difference between scarcity (3.55 ± 1.92) and abundance (3.61 ± 1.49) mindsets, *p* = 0.899. Please see Figure 6.

#### 4.2.3. Effects of Mindset and Social Norms on Norm Perception

Independent-samples *t*-tests were conducted respectively for high and low prosocial conditions to examine the effect of mindset on norm perception. The results indicated that under high prosocial condition, participants in scarcity mindset (77.54% ± 12.43%) perceived higher level of cooperation compared to those in abundance mindset (65.71% ± 20.72%), *t*(68) = 2.90, *p* = 0.005, Cohen’s *d* = 0.69. Under the low prosocial condition, there was no significant difference in norm perception between these two mindsets (Scarcity_(M ± SD)_ = 32.63% ± 26.14%, Abundance_(M ± SD)_ = 42.09% ± 23.24%), *t*(68) = −1.60, *p* = 0.114, Cohen’s *d* = −0.38.

#### 4.2.4. Combined Analysis of Experiments 2 and 3

To further investigate the generality of these effects, we combined data from Experiment 2 (descriptive norm context) and Experiment 3 (injunctive norm context) for a comprehensive analysis. A 2 (mindset: scarcity vs. abundance) × 2 (norm type: descriptive vs. injunctive) × 2 (prosocial level: high vs. low) mixed ANOVA was conducted on PGG contributions, with mindset and norm type as the between-subject factors, prosocial level as the within-subjects factor.

This three-way analysis yielded significant main effects of mindset, norm type, and prosocial level. Participants in the scarcity mindset (4.07 ± 1.41) contributed more money compared to those in the abundance mindset (3.14 ± 1.72), *F*(1, 120) = 15.34, *p* < 0.001, η_p_^2^ = 0.113. Contributions were also higher under injunctive norms (4.30 ± 1.46) compared to descriptive norms (2.72 ± 1.41), *F*(1, 120) = 41.07, *p* < 0.001, η_p_^2^ = 0.255. Additionally, participants contributed more in the high prosocial condition (4.38 ± 1.98) than in the low prosocial condition (2.84 ± 1.79), *F*(1, 120) = 103.66, *p* < 0.001, η_p_^2^ = 0.463.

The interaction effect between mindset and prosocial level was significant, *F*(1, 120) = 32.51, *p* < 0.001, η_p_^2^ = 0.213. Simple-effects analysis revealed that under high prosocial condition, participants in scarcity mindset (5.26 ± 1.61) contributed more than those in abundance mindset (3.49 ± 1.92), *p* < 0.001. However, under the low prosocial condition, there was no significant difference in scarcity mindset (2.88 ± 1.87) and in abundance mindset (2.80 ± 1.72), *p* = 0.801. No other significant interactions were found; *ps* > 0.21.

### 4.3. Discussion

In contrast to descriptive norms, which reflect what behaviors are typically performed, injunctive norms prescribe what behaviors are considered socially acceptable. Experiment 3 built upon this distinction by examining how injunctive norms, which dictate appropriate actions, influence cooperation across different mindsets.

As hypothesized, participants with a scarcity mindset showed a stronger tendency to cooperate in the PGG than those in an abundance mindset, replicating earlier findings. Injunctive norms shape individuals’ actions by appealing to their desire for social approval and recognition (Cialdini & Goldstein, 2004; McAuliffe et al., 2017; Schultz et al., 2007; Stok & de Ridder, 2019; Thomas et al., 2016). The heightened cooperation observed among participants under a scarcity mindset may reflect an increased motivation for group approval when resources are perceived as scarce.

Furthermore, the strength of prosocial norms had a significant influence on cooperation. Participants exposed to high prosocial norms contribute more than those in low prosocial condition, which aligns with previous research on the impact of norm strength on cooperative behavior (Bicchieri & Xiao, 2009; Camerer & Fehr, 2006). This finding reinforces the idea that stronger normative encouragement (i.e., high prosocial norms) universally promotes cooperative contributions.

Importantly, we identified a significant interaction between mindset and injunctive norms in shaping cooperation. More specifically, under the low prosocial condition, cooperation remained low regardless of participants’ mindset. However, under high prosocial conditions, those with a scarcity mindset contributed significantly more than those with an abundance mindset. This pattern supports Hypothesis 1, suggesting that injunctive norms, like descriptive norms, can influence cooperation, but the effects are stronger when prosocial norms are emphasized. The norm perception analysis could reveal the psychological mechanisms driving this interaction. Under the high prosocial condition, scarcity-mindset participants expected more cooperative behavior from others than did abundance-mindset participants. This optimistic expectation suggests that scarcity may lead individuals to overestimate others’ compliance with the norm and anticipate more prosocial group behavior, which in turn fosters higher contributions (Fischbacher et al., 2001).

Finally, the combined analysis of Experiments 2 and 3 revealed the moderating role of social norms in the relationship between mindset and cooperation across both normative contexts. Specifically, the interaction between scarcity mindset and prosocial norm strength emerged reliably under both descriptive and injunctive norms, offering robust support for Hypothesis 2. This convergence across experiments underscores the generalizability of the observed effects and highlights the critical role of social norms in shaping how individuals’ resource-related mindsets influence cooperative behavior.

## 5. General Discussion

This study, conducted across three experiments, examined how mindset and prosociality influence cooperation within descriptive and injunctive norm contexts. The results revealed that mindset exerted a significant impact on cooperation in both norm contexts, with the scarcity mindset promoting greater cooperation. Additionally, participants exhibited higher cooperation under high prosocial condition compared to low prosocial condition. Crucially, an interaction effect was observed between mindset and prosocial level: under the low prosocial condition, cooperation levels did not differ significantly between scarcity and abundance mindsets. However, under high prosocial condition, individuals with a scarcity mindset displayed significantly greater cooperation than those with an abundance mindset.

### 5.1. Scarcity Mindset Enhances Cooperation

The results of the three experiments align with previous research, showing a significant main effect of mindset on cooperation, with higher cooperation observed under the scarcity mindset. Previous studies have identified positive correlations between long-term scarcity experiences and prosocial behavior (Civai et al., 2022; Petersen et al., 2014). For instance, empirical research by Nie et al. (2020) found that individuals from resource-scarce regions were more likely to contribute in public goods game. Although participants in the current study did not experience actual material deprivation, the temporary activation of a scarcity mindset was sufficient to enhance subsequent cooperation. This suggests that the psychological effects of perceived scarcity can foster prosocial behavior, even in the absence of objective material deprivation.

One possible explanation for this effect is that scarcity heightens individuals’ reciprocal motivation. Previous research has found that individuals with a scarcity mindset are more inclined toward reciprocal cooperation in repeated interaction settings (Boyce-Jacino, 2017). Given that our study used a multi-round PGG, it is plausible that scarcity induced a stronger inclination to engage and reciprocate within the group, motivating individuals to cooperate even when such behavior carries some risk.

An alternative explanation involves increased risk preference associated with scarcity. Cooperation inherently entails risk, and numerous studies have suggested that scarcity can elevate individuals’ risk-taking tendencies in pursuit of greater potential rewards (Callan et al., 2011; Liang et al., 2021). However, cooperation in the PGG does not always yield greater returns; in fact, “free riding” could be a more profitable strategy. Therefore, while increased risk tolerance might contribute to the observed cooperation, it does not fully account for the behaviors observed under scarcity.

Some studies have discovered that scarcity might hinder cooperative behavior (Cui et al., 2022). According to the Conservation of Resources (COR) theory, acute resource depletion can trigger self-protection mechanisms, leading to aggressive or irrational behaviors (Hobfoll et al., 2018). Although these findings appear to contradict the present study, we argue that the perceived availability of the resources plays a critical role (Faber & Häusser, 2022). In previous studies, the resource perceived as scarce was also the one being allocated. In contrast, in our study, the resource for cooperation was not perceived as scarce but rather as accessible, potentially explaining the divergence in findings.

### 5.2. The Role of Social Norms in Modulating Scarcity Mindset and Cooperation

Our findings further demonstrated that when group cooperation levels were low, individuals—regardless of mindset—exhibited low cooperation. This outcome aligns with evidence showing that uncooperative behavior can quickly spread within groups and suppress others’ willingness to contribute (Fowler & Christakis, 2010). It has been proposed that while group cooperation depends on individual contributions, unconditional cooperation within a group can be costly for individuals (Ohtsuki, 2018; Thürmer et al., 2020). In situations of conflict, individuals tend to prioritize their own interests over those of the group. COR theory also maintains that individuals strive to acquire, conserve, and protect what they value (Hobfoll et al., 2018). Thus, in low prosocial condition, contributing involves personal risk, which can reduce cooperation.

In contrast, individuals with a scarcity mindset exhibited increased cooperation under high prosocial condition. This effect may be explained by the internalization of social norms. Individuals tend to internalize norms by observing the rewards or punishments associated with behavior (Gavrilets & Richerson, 2017; Gross & Vostroknutov, 2022). In our repeated PGG setting, individuals with a scarcity mindset appeared to align more closely with cooperative norms than their abundance-minded peers. We suppose that although both mindset groups could learn these norms, those in a scarcity mindset may have internalized them to a greater extent.

According to social cognitive theory of social class, individuals from lower socioeconomic status—who frequently experience scarcity—tend to adopt situational cognitive tendencies and interdependent social orientations (Kraus et al., 2012). These tendencies may render them more susceptible to external cues, leading those experiencing scarcity to adhere to group norms as a means of seeking social recognition and belonging, thereby reducing the discomfort associated with scarcity (Cannon et al., 2019). Recent neuroscience research suggests that a scarcity mindset can amplify empathic responses and prosocial intentions toward others (Osman et al., 2018), supporting the notion that scarcity increase sensitivity to normative expectations and concern for others. Our findings reflect a short-term analog for the long-term adaptations observed in individuals from lower SES backgrounds.

Moreover, strong prosocial norms may prompt individuals to rely more on intuitive thinking, which in turn fosters cooperation. Prior studies indicate that intuitive responses are more likely to promote cooperative behavior when such behavior aligns with shared expectations (Rand et al., 2012, 2016). Bear and Rand (2016) argue that this tendency reflects the internalization of norms shaped by previous exposure to cooperative environments. Capraro (2024) further emphasizes that intuitive decisions often rely on simple heuristics—such as avoiding harm and rejecting unfairness—that promote prosocial choices. Individuals with a scarcity mindset, who are especially sensitive to contextual cues (Kraus et al., 2012; Cannon et al., 2019), may be more attuned to these normative signals. Under high prosocial norms, they may follow intuitive impulses to cooperate, aligning their behavior with group expectations.

The interaction between mindset and prosocial norms highlights that prosocial tendencies under scarcity are context-dependent. Scarcity increases the willingness to cooperate when others are cooperating, but it may fail to do so in predominantly non-cooperative environments. This finding enhances norm theory by emphasizing how personal resource mindset can moderate the influence of social norms. Additionally, it enriches scarcity theory: beyond cognitive effects like narrowed attention, scarcity has social effects—it predisposes individuals to adapt to prevailing social norms. Future research could extend this framework by exploring whether interventions that combine scarcity cues with positive normative information can reliably enhance collective outcomes.

### 5.3. Limitation and Future Directions

The current research has several limitations: First, the ecological validity of our design warrants improvement. Participants were informed that other players’ decision were pre-recorded, which enabled rigorous control over experimental conditions. However, this setup reduced the realism of social interactions and may limit the generalizability of our findings to real-world cooperative settings.

Second, the generalizability of the findings is limited by the characteristics of the participant sample, which primarily consisted of Chinese female college students. Although prior research has found minimal gender differences in cooperation (Balliet et al., 2011; Spadaro et al., 2023), the overrepresentation of women may still influence the observed effects. Additionally, participants were drawn from a collectivist cultural background, which may shape responses to social norms differently than in individualistic cultures. Future research should examine whether the current findings replicate across more diverse gender and cultural contexts.

Third, the mechanisms underlying norm influence cooperation require further exploration. Previous research suggests that individuals adhere to descriptive norms due to informational motivation (i.e., a desire to make the correct choice), while injunctive norms are followed due to affiliative motivation. Future research should examine these motivational processes to clarify how they interact with scarcity mindset.

Finally, although we distinguished between descriptive and injunctive norms, the study did not include direct checks to verify participants’ understanding of these norm types. Moreover, the proposed internalization mechanism was not directly assessed. As norm internalization is latent and difficult to infer from behavior alone, future research should employ more targeted methods to validate both norm perception and internalization, especially under scarcity conditions.

## 6. Conclusions

This study examined the impact of scarcity mindset and social norms on cooperation, leading to the following conclusions: Individuals with a scarcity mindset exhibited higher cooperation than those with an abundance mindset. The level of prosocial norms significantly influences cooperation, with higher prosocial norms fostering greater cooperation. Notably, under low prosocial norms, cooperation did not differ significantly between mindsets; however, scarcity-minded individuals demonstrated significantly greater cooperation under high prosocial norms. There results suggest that a scarcity mindset heightens individuals’ responsiveness to prosocial norms, reinforcing the view that context critically shapes how scarcity impacts social behavior.

## Data Availability

The dataset and analysis code for this study are available at the following link: https://osf.io/v3a9x/?view_only=a0031efe55144e1bbc6652b89c917f12 (accessed 16 November 2024).
